# Derivation of an electronic frailty index for predicting short‐term mortality in heart failure: a machine learning approach

**DOI:** 10.1002/ehf2.13358

**Published:** 2021-06-03

**Authors:** Chengsheng Ju, Jiandong Zhou, Sharen Lee, Martin Sebastian Tan, Tong Liu, George Bazoukis, Kamalan Jeevaratnam, Esther W.Y. Chan, Ian Chi Kei Wong, Li Wei, Qingpeng Zhang, Gary Tse

**Affiliations:** ^1^ Research Department of Practice and Policy, School of Pharmacy University College London London UK; ^2^ School of Data Science City University of Hong Kong Hong Kong SAR China; ^3^ Cardiovascular Analytics Group, Laboratory of Cardiovascular Physiology, LKS Institute of Health Sciences Chinese University of Hong Kong Hong Kong SAR China; ^4^ Faculty of Arts and Science University of Toronto Toronto Ontario Canada; ^5^ Tianjin Key Laboratory of Ionic‐Molecular Function of Cardiovascular Disease, Department of Cardiology, Tianjin Institute of Cardiology Second Hospital of Tianjin Medical University Tianjin China; ^6^ Second Department of Cardiology Evangelismos General Hospital Athens Greece; ^7^ Faculty of Health and Medical Sciences University of Surrey Guildford UK; ^8^ Centre for Safe Medication Practice and Research, Department of Pharmacology and Pharmacy The University of Hong Kong Hong Kong SAR China

**Keywords:** Frailty index, Heart failure, Mortality, Inflammation, Nutrition, Machine learning

## Abstract

**Aims:**

Frailty may be found in heart failure patients especially in the elderly and is associated with a poor prognosis. However, assessment of frailty status is time‐consuming, and the electronic frailty indices developed using health records have served as useful surrogates. We hypothesized that an electronic frailty index developed using machine learning can improve short‐term mortality prediction in patients with heart failure.

**Methods and results:**

This was a retrospective observational study that included patients admitted to nine public hospitals for heart failure from Hong Kong between 2013 and 2017. Age, sex, variables in the modified frailty index, Deyo's Charlson co‐morbidity index (≥2), neutrophil‐to‐lymphocyte ratio (NLR), and prognostic nutritional index at baseline were analysed. Gradient boosting, which is a supervised sequential ensemble learning algorithm with weak prediction submodels (typically decision trees), was applied to predict mortality. Variables were ranked in the order of importance with a total score of 100 and used to build the frailty models. Comparisons were made with decision tree and multivariable logistic regression. A total of 8893 patients (median: age 81, Q1–Q3: 71–87 years old) were included, in whom 9% had 30 day mortality and 17% had 90 day mortality. Prognostic nutritional index, age, and NLR were the most important variables predicting 30 day mortality (importance score: 37.4, 32.1, and 20.5, respectively) and 90 day mortality (importance score: 35.3, 36.3, and 14.6, respectively). Gradient boosting significantly outperformed decision tree and multivariable logistic regression. The area under the curve from a five‐fold cross validation was 0.90 for gradient boosting and 0.87 and 0.86 for decision tree and logistic regression in predicting 30 day mortality. For the prediction of 90 day mortality, the area under the curve was 0.92, 0.89, and 0.86 for gradient boosting, decision tree, and logistic regression, respectively.

**Conclusions:**

The electronic frailty index based on co‐morbidities, inflammation, and nutrition information can readily predict mortality outcomes. Their predictive performances were significantly improved by gradient boosting techniques.

## Introduction

Frailty refers to a reduced physiological reserve leading to an impairment in resilience from physical distress. Compared with highly functional community‐dwelling elders, frail older adults are more likely to experience falls and disability, contributing to frequent hospitalization and premature death.^1^ Conventional evaluation of frailty relies on physical examination. However, this precludes its calculation using administrative data such as electronic health records. Recently, a claims‐based frailty scoring system has been validated against Fried and colleges' frailty phenotype using a claim database in the USA.^2^, ^3^, ^4^ These electronic frailty indices do not normally include measures of chronic inflammation or nutrition status, which are both closely related to frailty syndrome and are strong determinants of adverse outcomes such as mortality.^5^, ^6^

Heart failure is a complex syndrome characterized by high prevalence in older patients and poor prognosis.^7^, ^8^ Heart failure and frailty have an overlapping phenotype, and their co‐existence is common.^9^ Given these associations, there has been several studies exploring the intersections between heart failure and frailty.^9^ Importantly, frailty has been recognized as a major prognostic indicator of heart failure, in which patients with concurrent frailty and heart failure have increased risks of hospitalizations and mortality.^10^

Furthermore, inflammation and nutrition status are known independent predictors of heart failure outcomes.^11^, ^12^, ^13^ Inflammation has a pivotal role in the pathogenesis of heart failure.^14^ It can trigger cardiac remodelling and dysfunction that further induce cardiomyocyte damage that underlies heart failure.^15^ Moreover, it has been proposed that co‐morbidities, such as diabetes and obesity, can induce a systemic pro‐inflammatory state that drives the myocardial structural and functional alterations in heart failure.^16^, ^17^ Conversely, inflammation can also be a consequence of established heart failure via the mechanisms of increased wall stress on endothelial cells, cell death, and oxidative stress.^18^ In this regard, inflammation and heart failure are interconnected and mutually inducing. Elevated pro‐inflammatory cytokines were found to associate with worse clinical outcomes in patients with heart failure,^19^, ^20^ and some studies have demonstrated that neutrophil‐to‐lymphocyte ratio (NLR) can be used as a prognostic factor for heart failure.^21^ Similar to inflammation, malnutrition is also an independent risk factor and prognostic factor for heart failure.^22^, ^23^ Various nutritional metrics, including prognostic nutritional index (PNI), associate well with the survival outcomes.^24^, ^25^ Both factors possess important predictive values for clinical outcomes in patients with heart failure.^26^, ^27^, ^28^ Despite the multiple associations between frailty, heart failure, nutrition status, and inflammation, whether incorporating the measures of nutrition status and inflammation into the existing frailty index can enhance its predictive value on outcomes of heart failure remains unknown.

Machine learning techniques have gained popularity in medical research. Specifically, gradient boosting has recently been explored as a method to predict adverse outcomes in heart failure.^29^ In this study, using a large cohort of patients with heart failure, we tested the hypothesis that incorporating NLR and PNI into an electronic frailty index using gradient boosting, a machine learning approach will improve prediction for short‐term mortality risks.

## Methods

### Data source and study population

This study received Ethical Approval from the local Ethics Committee. This is a retrospective cohort study nested within the territory‐wide Clinical Data Analysis and Reporting System, an electronic health record system managed by the Hong Kong Hospital Authority since 1995. The database included over seven million Hong Kong residents and has been used for producing high‐quality clinical studies.^30^, ^31^

Patient information was de‐identified with pseudo‐identity numbers. Clinical data available from Clinical Data Analysis and Reporting System include demographic information, diagnosis, procedure, prescription, laboratory test results, admission/discharge information, and death information.

​

The inclusion criterion was patient admitted to any of the nine local hospitals during a 4 year period between July 2013 and July 2017 with a principal diagnosis of heart failure. The diagnosis of heart failure was defined as having a record with the International Classification of Diseases, Ninth Revision, Clinical Modification (ICD‐9 CM) codes of 428.X.

### Study variables

Variables that were previously included in the modified frailty index^4^ were identified from the relevant ICD‐9 codes. These include depression, Parkinson's disease, arthritis, paranoia, chronic skin ulcer, pneumonia, falls, skin and subcutaneous tissue infection, mycoses, gouty arthropathy, and urinary tract infection. Laboratory test results on the measures of albumin level, neutrophil, and lymphocyte counts were extracted to calculate inflammatory and nutritional indices. NLR was given by the ratio of peripheral neutrophil count/mm^3^ to peripheral lymphocyte count/mm^3^. PNI was calculated by 10 × serum albumin value (g/dL) + 0.005 × peripheral lymphocyte count/mm^3^. NLR and PNI estimates nearest to the admission time of the first heart failure related hospitalization of the patients were used in the analysis. Baseline Deyo's Charlson co‐morbidity index incorporating 17 major medical conditions was also included as a single score.^32^ A comparison of the included variables used in the modified frailty index, Charlson Deyo's Charlson co‐morbidity index, and our electronic frailty index is shown in the Supporting Information, *Table* *S1*.

### Outcomes and statistical analysis

The primary outcomes were 30 day and 90 day mortality, from the date of the first heart failure related‐hospitalization of the patients. The outcome of 30 day mortality is binary and equal to 1 for mortality within 30 days and 0 otherwise, and the same for 90 day mortality outcome. Continuous variables were presented as median (interquartile range [IQR]), and categorical variables were presented as count (%). The Mann–Whitney *U* test was used to compare continuous variables. The *χ* ^2^ test with Yates' correction was used for 2 × 2 contingency data, and Pearson's *χ* ^2^ test was used for contingency data for variables with more than two categories. To identify significant risk factors associated with 30 day and 90 day mortality, univariate logistic regression was used to determine odds ratios and 95% confidence intervals. Significant variables from the univariable logistic regression (*P* < 0.05) were further included in multivariable logistic regression to build the frailty model.

The idea of frailty is the cumulative deficits, each of which in isolation may not exert significant effects. To test this idea, we conducted an additional multivariable logistic regression analysis incorporating all risk variables, including the non‐significant variables from univariable logistic regression. Finally, to demonstrate the utility of NLR and PNI, both variables were excluded in sensitivity analysis to examine the effects on evaluation metrics.

A two‐sided α of <0.05 was considered statistically significant. All statistical analyses were performed using RStudio software (Version: 1.1.456).

### Machine learning model development

Gradient boosting is a typical type of machine learning boosting, relying on the intuition that the best possible next model, when sequentially combined with previous weak models (e.g. decision trees) in a stage‐wise fashion, is able to minimize the overall prediction error measured by performance evaluators, for example, precision, recall, and the area under the curve (AUC). Weaker learning models are fitted through loss gradient minimization with gradient descent optimization algorithm.^33^ This method was used for mortality prediction in heart failure based on administrative claims with electronic health records.^29^ Variable importance ranking was generated to construct a machine learning based risk score for mortality prediction. Partial dependence plots were provided as low‐dimensional graphical renderings of marginal effects to assist in the interpretation of relationships between the most important variables and the mortality outcome. A five‐fold cross validation was performed to compare the performance in terms of precision, recall, and AUC of the gradient boosting model with decision tree model and logistic regression model. The R packages, *gbm* (Version 2.1.5) and *ggplot2* (Version 3.3.2), were used to generate the mortality prediction results.

## Results

In our heart failure cohort (*n* = 8893), the median age was 81 (IQR 71–87) years, and 45% (*n* = 4027) were men. The baseline characteristics, individual variables included in the modified frailty index, inflammatory and nutritional indices between the patients died within 90 days and the patients without 90 day mortality are shown in *Table* *1*. The median cell counts for lymphocytes was 1.2*10^9^/L and for neutrophils was 5.4*10^9^/L, yielding an NLR of 4.4 (IQR 2.7–7.8). Albumin took a median level of 37.8 g/L, yielding a PNI of 44.0 (IQR 39.8–48.5) (PNI, given by 10 × serum albumin value (g/dL) + 0.005 × peripheral lymphocyte count (per mm^3^)).

**Table 1:** Baseline characteristics of the heart failure cohort

	90‐day mortality *N* = 1472	No mortality *N* = 7421	*P*‐value
**Gender**			
Male (%)	639 (43.4%)	3388 (45.7%)	0.114
**Age**	84.4 [77.5–90.1]	80.0 [70.1–85.9]	**<0.001**
**Modified frailty index**			
Depression	1 (0.1%)	15 (0.2%)	0.276
Parkinson's disease	18 (1.2%)	55 (0.7%)	0.061
Arthritis	7 (0.5%)	33 (0.4%)	0.872
Paranoia	0 (0.0%)	7 (0.1%)	0.239
Skin ulcer	35 (2.4%)	71 (1.0%)	**<0.001**
Pneumonia	549 (37.3%)	1492 (20.1%)	**<0.001**
Falls	36 (2.5%)	154 (2.08%)	0.369
Skin and soft tissue infection	17 (1.2%)	82 (1.1%)	0.868
Mycoses	2 (0.1%)	17 (0.2%)	0.479
Gouty arthropathy	87 (5.9%)	354 (4.8%)	0.066
UTI	209 (14.2%)	560 (7.6%)	**<0.001**
Charlson score ≥2	775 (52.7%)	697 (47.4%)	**<0.001**
**Inflammatory and nutritional indices**			
PNI	41.5 [37.0–46.0]	44.5 [40.4–48.8]	**<0.001**
NLR	5.5 [3.1–9.8]	4.2 [2.6–7.4]	**<0.001**

NLR, neutrophil‐to‐lymphocyte ratio; PNI, prognostic nutritional index; UTI, urinary tract infection.

Values in bold indicate *P* < 0.05.

### Predictors of adverse outcomes and frailty model

Of the 8893 patients with heart failure, 758 patients died within 30 days (9%), and 1472 died within 90 days (17%) of admission. The findings of univariate logistic regression are reported in the Supporting Information, *Table* *S2*. Age, chronic skin ulcers, pneumonia, urinary tract infection, NLR, and PNI were significant predictors of 30 day mortality (Supporting Information, *Table* *S2*, left). For 90 day mortality, the same variables that predicted 30 day mortality, as well as Charlson score ≥2 were significant predictors (Supporting Information, *Table* *S2*, right). Subsequently, the significant variables from the univariable analysis were included in multivariable logistic regression. The results of multivariable logistic regression for 30 day and 90 day mortality prediction with all variables are reported in the Supporting Information, *Tables* *S3* and *S4*, respectively. Age, pneumonia, UTI, PNI, and NLR remained significant predictors of both 30 day and 90 day mortality (*P* < 0.05).

### Gradient boosting learning results and frailty score

Five‐fold cross validation experiments were conducted with gradient boosting learning. The key to gradient boosting is to set the target outcomes to minimize the overall error in relation to precision, recall, and AUC. In this way, the gradient boosting model sequentially adds weak decision tree learning models to the ensemble where subsequent models correct the prediction errors of prior models (Supporting Information, *Figure* *S1*), from which we can see that probability of 30 day mortality and 90 day mortality increases drastically as age grows above 80 years old. Specifically, predictions given by the sequential models that are close to the actual outcome should reduce the overall error, and the process continues until minimized total prediction error is achieved.

A total of 1400 and 1500 trees for 30 day and 90 day mortality prediction were assigned, respectively. The optimal tree number was identified using sensitivity analysis by plotting the value of the out‐of‐bag (OOB) error rate according to the number of trees within the forest (Supporting Information, *Figure* *S2*). OOB samples are those samples that are not included in the bootstrap samples. Original training data are randomly sampled‐with‐replacement generating small subsets of data, also known as bootstrap samples. These bootstrap samples are then fed as training data to the forest model. The OOB approach was used for selecting the optimal tree number of the forest model in which four‐fifths (as training) of the data were used for constructing the predictive classifier, while the remaining was used for evaluating the performance of the forest model. Tree depth was set at 1 for both 30 day and 90 day mortality prediction according to tree depth parameter tuning (Supporting Information, *Figure* *S3*). The variable importance is reported in *Table* *2* and is used for building the frailty score.

**Table 2:** Variable importance for 30‐day and 90‐day mortality prediction with gradient boosting learning

30‐day mortality		90‐day mortality	
Variable	Importance	Variable	Importance
PNI	37.45	Age	36.25
Age	32.11	PNI	35.28
NLR	20.46	NLR	14.59
Pneumonia	6.04	Pneumonia	7.05
Skin ulcer	1.16	UTI	2.29
UTI	1.03	Skin ulcer	1.43
Parkinson's disease	0.49	Male sex	0.57
Male sex	0.45	Falls	0.48
Skin and soft tissue infection	0.25	Parkinson's disease	0.46
Gout	0.23	Charlson score ≥2	0.46
Falls	0.19	Arthritis	0.35
Charlson score ≥2	0.13	Skin and soft tissue infection	0.34
Arthritis	0.01	Gout	0.31
Mycoses	0.01	Mycoses	0.15
Depression	0	Depression	0
Paranoia	0	Paranoia	0

NLR, neutrophil‐to‐lymphocyte ratio; PNI, prognostic nutritional index; UTI, urinary tract infection.

**Figure 1:**
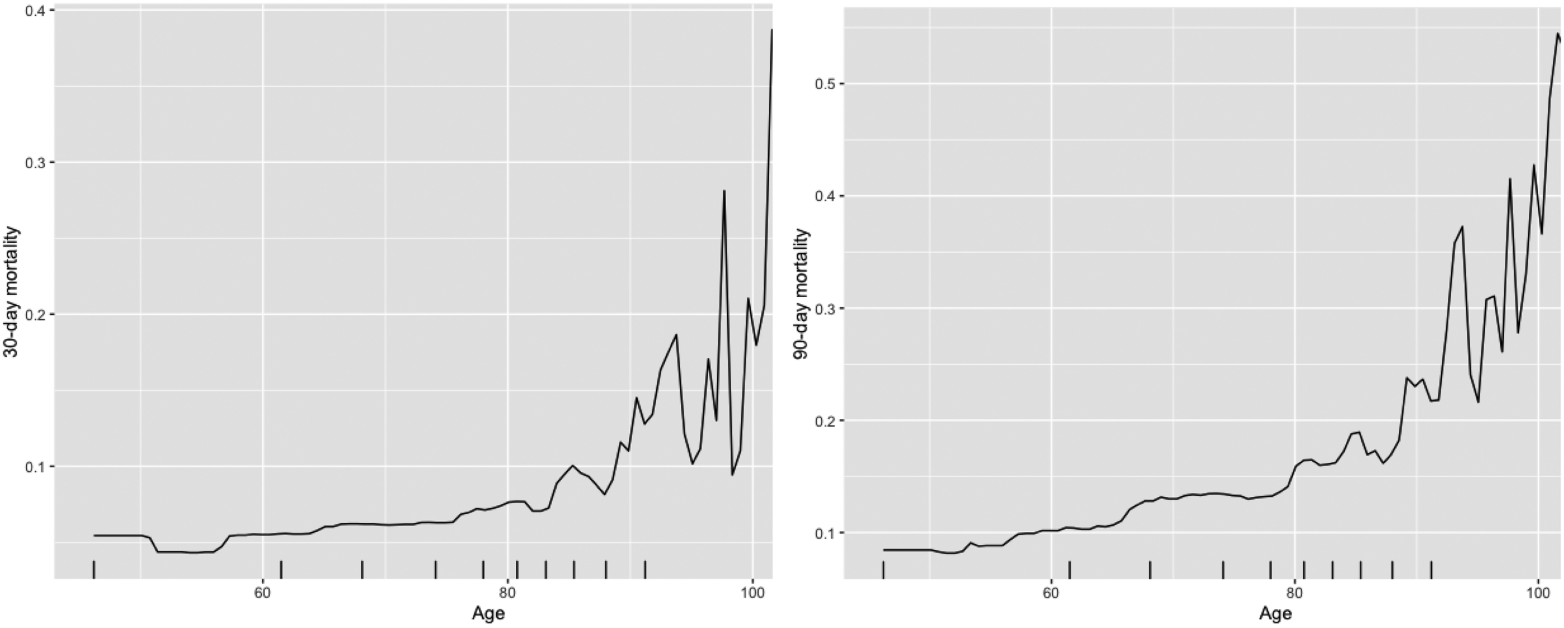
Partial dependence of patient age for 30 day (left) and 90 day (right) mortality risk probability prediction.

**Figure 2:**
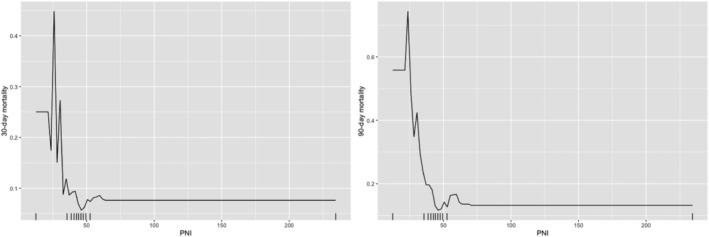
Partial dependence of PNI for 30 day (left) and 90 day (right) mortality risk probability prediction.

**Figure 3:**
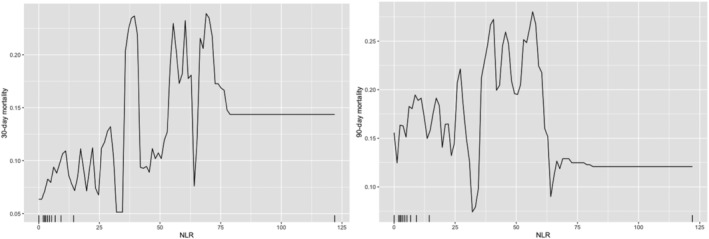
Partial dependence of NLR for 30 day (left) and 90 day (right) mortality risk probability prediction.

The partial dependence relationships of the highest variable importance values for mortality prediction were also identified using gradient boosting learning. The probabilities of 30 day and 90 day mortality both increase as patient becomes older, and it increases sharply when patients are older than 80 (*Figure* *1*). For PNI, the likelihood of mortality decreases sharply as PNI increases from 0 to 20 and remains almost constant when PNI increases beyond 65 (30 day mortality) or 70 (90 day mortality) (*Figure* *2*). There is a non‐linear relationship between NLR and 30 day and 90 day mortality (*Figure* *3*). Patients with pneumonia has high probability of mortality, 24% for 30 day mortality, and 14% for 90 day mortality.

Comparative analyses of gradient boosting learning model, decision tree model, and multivariable logistic regression model for 30 day and 90 day mortality prediction are reported in the Supporting Information, *Table* *S5* with five‐fold cross validation. Gradient boosting learning shows the best performance in prediction, recall, and AUC evaluation metrics.

The results from the sensitivity analysis excluding NLR and PNI are shown in the Supporting Information, *Appendix* *S2*. The optimum tree numbers are shown in the Supporting Information, *Figure* *S4*. Without NLR and PNI, age became the most important variable for predicting both 30 day and 90 day mortality (Supporting Information, *Table* *S6*; *Figure* *S5*), and some evaluation metrics were lower, but others were not affected (Supporting Information, *Table* *S7*). Five cross validations indicate that the machine learning model maintains comparable prediction performance as the case with NLR and PNI to predict 30 day mortality (precision = 0.90, recall = 0.89, and AUC = 0.90) and 90 day mortality (precision = 0.91, recall = 0.91, and AUC = 0.90).

## Discussion

The main findings of this study are that (i) baseline PNI, NLR, and age at heart failure hospitalization in the modified electronic frailty index were the most predictive variables for the short‐term mortality outcomes in heart failure patients, and (ii) non‐linear partial dependence relationships between these predictors and outcomes were observed.

We developed a modified electronic frailty model after incorporating the inflammatory and nutritional indices into the conventional frailty scoring system based on the value of importance of each variable generated from gradient boosting learning model. Compared with multivariable logistic regression and decision tree, gradient boosting learning techniques improved the predictive performance of our frailty model. To enhance mortality prediction by capturing the non‐linear pattern within characteristics, we develop an interpretable machine learning model based on gradient boosting machine.^34^ Machine learning models can be fitted to data individually or combined in an ensemble, resulting in an efficient combination of simple individual learning models that together create a more powerful model.^35^

In this study, significant risk factors to predict 30 day and 90 day mortality are efficiently identified with gradient boosting learning model. The obtained rankings of important variables for mortality prediction can be used as an electronic heart failure frailty scoring tool for clinical use. The efficient identification of partial dependence for predictive variables provides more refined estimation of the likelihood of mortality. For example, effective estimations about the patient's mortality probability based on characteristics of smaller PNI, older age, larger NLR (below 60 or so), and pneumonia. All of these variables were associated with impaired mobility. Of these factors, pneumonia is a common nosocomial condition that also confers a significantly higher risk of 30 day post‐admission mortality.^36^ In addition, we extensively conduct the analysis without PNI and NLR, and the results are provided in the supporting information. Variable importance ranking for 30 day identifies important variables age, pneumonia, skin ulcer, UTI, Parkinson's, gout, and male sex to predict 30 day mortality, while variables age, pneumonia, UTI, skin ulcer, Parkinson's, and Carlson score to predict 90 day mortality.

Heart failure has been recognized as predominately a syndrome that affects the geriatric population, with over 50% of incidence and 60% of heart failure‐associated mortality occurring in the population over 75 years old.^37^ Age at diagnosis is also one of the most significant prognostic factors for subsequent survival.^38^ In our cohort, the median age was 81 years old, and the risk of the short‐term mortality increased strikingly in those aged over 80. Age was ranked as the most important variable to predict 90 day mortality and the second most important variable for 30 day mortality. In 2011, it was reported that the 1 year mortality rates increased sharply from 20% to over 30% in patients 75–84 years and over 40% in patients aged over 85 years.^39^ The high prevalence of important risk factors, such as hypertension and ischaemic heart disease, leads to the increasing incidence of heart failure in older patients.^40^ Moreover, the survival outcomes of heart failure are closely related to the unfavourable age‐associated changes in cardiovascular structure and function, which compromise cardiac reverse capacity.^41^ Therefore, it is not surprising to observe the strong prognostic value of age in our frailty model.

The frailty index was based on the concept that frailty is caused by the accumulation of health deficits.^42^ The frailty state itself is considered as an individual variable that can predict mortality,^43^ even independently of age in different settings.^44^ The first electronic frailty index developed by Segal *et al*. was based on the same concept, in which the candidate variables were selected based on their potential correlation with the frailty state rather than mortality directly.^4^ Therefore, the individual variables in the frailty index might not associate well with the mortality outcome, and the deficits cumulatively lead to an increased risk of mortality.

The specific value of frailty in heart failure cohort has been examined by many studies. A recent meta‐analysis has confirmed the association between pre‐frailty or frailty state and the worse clinical outcomes in patients with heart failure.^45^, ^46^, ^47^ Indeed, recent guidelines have recommended the assessment of frailty status in heart failure patients to aid risk stratification.^48^ The Identification of Senior At Risk scale is another frailty screening tool that can predict 30 day mortality in older patients with acute heart failure.^49^ Among the current literature, a few studies utilized frailty indices and reached similar conclusions to other studies in which frailty was assessed as a phenotype,^50^, ^51^ and there is no consensus which method is more suitable in the cohort of heart failure patients.^45^ The variables included in the various frailty indices used for heart failure were also largely different. A study in the UK combined the frailty index and nutritional index and found an improved prognostic power compared with the conventional frailty index, suggesting that nutrition and frailty are correlated but also remained as independent prognostic factors.^51^ No previous study has attempted to incorporate inflammatory measures into frailty indices for heart failure prognosis despite the strong pathophysiological associations between these concepts.^52^

### Strength and limitations

To the best of our knowledge, this is the first study incorporating both the inflammatory marker and nutritional index into the conventional frailty index. The indices used in this study, NLR and PNI, can be easily calculated and incorporated into the decision‐making process in the clinical setting. We utilized a large patient cohort that is homogeneously Chinese from a real‐world database, and the final frailty score was derived from a machine learning model, which was shown to have a better performance than the baseline multivariate logistic regression for mortality prediction.

There are some limitations to our study. Firstly, this is a multicentre study conducted in Hong Kong, and external validation of our results using data from other countries is needed. Secondly, our study did not include information on the treatment prescribed during the acute phase and the postadmission period, which may affect the survival outcomes in patients. Thirdly, we cannot distinguish between subtypes of heart failure, such as heart failure with reduced rejection fraction or heart failure with reduced rejection fraction, as we do not have echocardiographic or relevant diagnostic codes available in our database.^53^ Moreover, there are some challenges of machine learning in clinical setting. Our model was developed specifically using the variables available in the local database and may have generalizability issue in other clinical settings if these variables were measured differently. Furthermore, the relative predictive values of the variables in our model might be difficult to interpret in regard to their biological associations with the mortality due to the opaque nature of the machine‐learning techniques. Nevertheless, a previous systematic review and meta‐analysis found that machine learning techniques can aid the diagnosis, management, and prediction of outcomes in heart failure patients.^54^ Nevertheless, frailty alone is a strong predictor of mortality, although it requires a thorough assessment that may be time‐consuming.^55^ Our study complements previous findings that electronic frailty indices are useful for risk prediction in other settings.^56^, ^57^, ^58^

## Conclusions

In this study, we created an electronic frailty index that included co‐morbidity information, inflammatory, and nutritional indices. This was then used for short‐term mortality prediction in heart failure. Given that these variables can be determined or calculated automatically, their incorporation into clinical risk scores or prediction rules will facilitate clinicians to perform risk stratification more readily. Further prospective studies are warranted to validate the present model by combining other more comprehensive and complex inflammatory, nutritional, and frailty assessment tools to confirm its predictive power for clinical use.

## Conflict of interest

All authors declare no conflict of interest.

## Supporting information


**Table S1.** Variables used in the modified frailty index proposed by Segal et al. (2017), Deyo‐Charlson comorbidity index (1992), and our electronic frailty index.
**Table S2.** Univariable logistic regression for 30‐day and 90‐day mortality.
**Table S3.** Multivariable logistic regression for 30‐day mortality.
**Table S4.** Multivariable logistic regression for 90‐day mortality.
**Table S5.** Five‐fold cross validation model performance for 30‐day and 90‐day mortality prediction.
**Figure S1.** Sequential ensemble concept of gradient boosting learning model.
**Figure S2.** Optimal iteration tree number of gradient boosting learning model for 30‐day and 90‐day mortality prediction.
**Figure S3.** Tree in‐depth parameter selection for 30‐day and 90‐day mortality prediction.

**Table S6.** Variable importance for 30‐day and 90‐day mortality prediction with gradient boosting learning without NLR and PNI.
**Table S7.** Five‐fold cross validation model performance for 30‐day and 90‐day mortality prediction without NLR and PNI.
**Figure S5**. Variable importance ranking for 30‐day and 90‐day mortality prediction without NLR and PNI.
