# Supplementary material for: Derivation of an electronic frailty index for predicting short‐term mortality in heart failure: a machine learning approach
Source: ESC Heart Fail. 2021 Jun 3;8(4):2837–45. doi: 10.1002/ehf2.13358 (PMC8318426; doi:10.1002/ehf2.13358)
Supplement: Supplementary file 2 — Table S6. Variable importance for 30‐day and 90‐day mortality prediction with gradient boosting learning without NLR and PNI. Table S7. Five‐fold cross validation model performance for 30‐day and 90‐day mortality prediction without NLR and PNI. Figure S5. Variable importance ranking for 30‐day and 90‐day mortality prediction without NLR and PNI. [file EHF2-8-2837-s002.docx]

**Supplementary Appendix 2: Sensitivity Analysis by excluding NLR and PNI**

**Table S6.** Variable importance for 30-day and 90-day mortality prediction with gradient boosting learning without NLR and PNI

| **30-day mortality** | | **90-day mortality** | |
| --- | --- | --- | --- |
| **Variable** | **Importance** | **Variable** | **Importance** |
| Age | 74.26 | Age | 70.79 |
| Pneumonia | 10.31 | Pneumonia | 11.25 |
| Skin.ulcer | 4.37 | UTI | 4.33 |
| UTI | 2.71 | Skin.ulcer | 3.67 |
| Parkinson’s | 2.56 | Parkinson’s | 1.67 |
| Male.sex | 1.36 | Charlson.Score>=2 | 1.62 |
| Gout | 1.22 | Male.sex | 1.61 |
| Falls | 1.03 | Falls | 1.44 |
| Soft.infection | 0.82 | Arthritis | 1.35 |
| Charlson.Score>=2 | 0.80 | Gout | 1.32 |
| Arthritis | 0.52 | Soft.infection | 0.76 |
| Mycoses | 0.05 | Mycoses | 0.14 |
| Depression | 0 | Depression | 0.07 |
| Paranoia | 0 | Paranoia | 0 |

UTI, urinary tract infection.

**Table S7.** Five-fold cross validation model performance for 30-day and 90-day mortality prediction without NLR and PNI

AUC: area under the curve; CI: confidence interval;

| **Model** | **30-day mortality** | | | **90-day mortality** | | |
| --- | --- | --- | --- | --- | --- | --- |
|  | **Precision** | **Recall** | **AUC [95% CI]** | **Precision** | **Recall** | **AUC [95% CI]** |
| Gradient boosting | 0.90 | 0.89 | 0.90[0.87,0.92] | 0.91 | 0.91 | 0.90[0.88,0.93] |
| Decision tree | 0.89 | 0.86 | 0.87[0.85,0.89] | 0.89 | 0.91 | 0.88[0.86,0.92] |
| Logistic regression | 0.86 | 0.84 | 0.86[0.81,0.88] | 0.82 | 0.82 | 0.86[0.82,0.87] |

AUC, area under the curve.

**
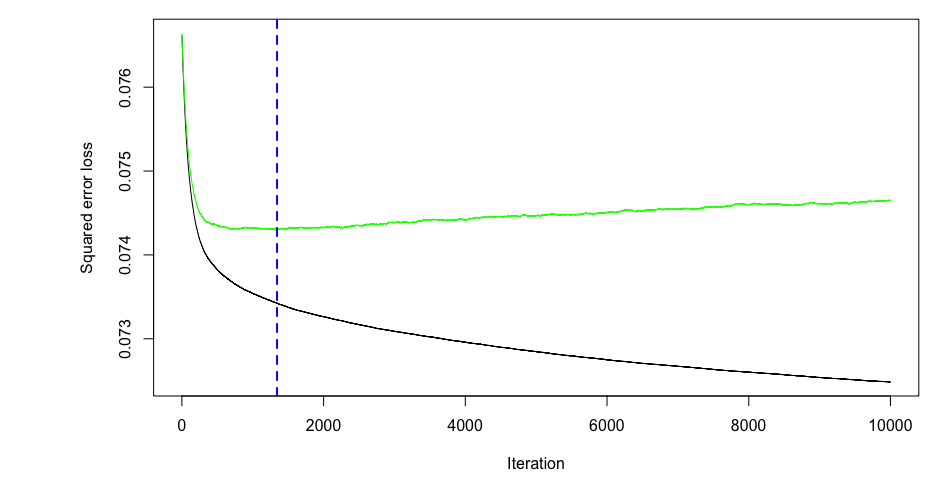

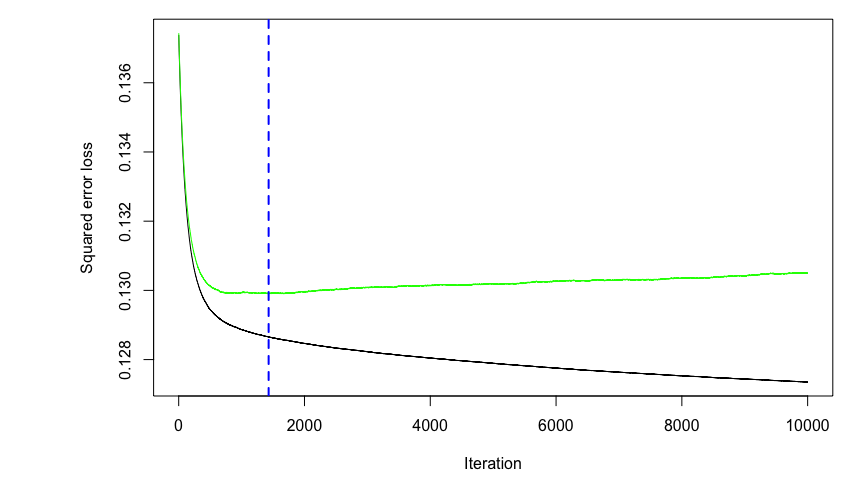
**

30-day mortality 90-day mortality

**Figure S4**. Optimal iteration tree number of gradient boosting learning model for 30-day and 90-day mortality prediction without NLR and PNI

**
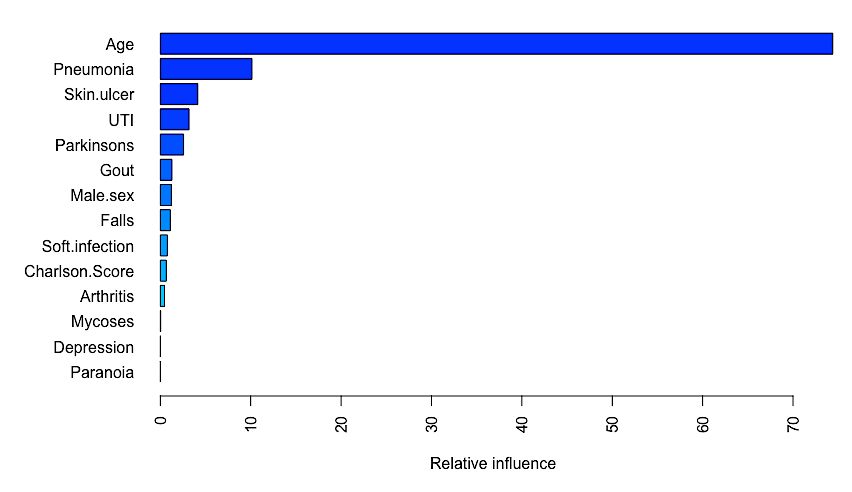

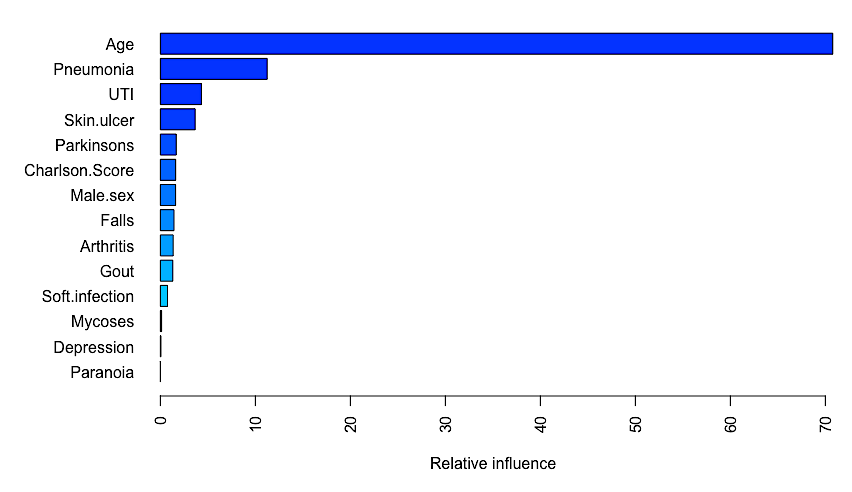
**

**Figure S5**. Variable importance ranking for 30-day and 90-day mortality prediction without NLR and PNI
